# Ubiquitination is involved in PKC-mediated degradation of cell surface Kv1.5 channels

**DOI:** 10.1016/j.jbc.2024.107483

**Published:** 2024-06-17

**Authors:** Ananya Chakraborty, Amanda Paynter, Mark Szendrey, James D. Cornwell, Wentao Li, Jun Guo, Tonghua Yang, Yuan Du, Tingzhong Wang, Shetuan Zhang

**Affiliations:** Department of Biomedical and Molecular Sciences, Queen’s University, Kingston, ON, Canada

**Keywords:** patch clamp, protein kinase C, ubiquitination, voltage-gated potassium channel

## Abstract

The voltage-gated Kv1.5 potassium channel, conducting the ultra-rapid delayed rectifier K^+^ current (I_Kur_) in human cells, plays important roles in the repolarization of atrial action potentials and regulation of the vascular tone. We previously reported that activation of protein kinase C (PKC) by phorbol 12-myristate 13-acetate (PMA) induces endocytic degradation of cell-surface Kv1.5 channels, and a point mutation removing the phosphorylation site, T15A, in the N terminus of Kv1.5 abolished the PMA-effect. In the present study, using mutagenesis, patch clamp recording, Western blot analysis, and immunocytochemical staining, we demonstrate that ubiquitination is involved in the PMA-mediated degradation of mature Kv1.5 channels. Since the expression of the Kv1.4 channel is unaffected by PMA treatment, we swapped the N- and/or C-termini between Kv1.5 and Kv1.4. We found that the N-terminus alone did not but both N- and C-termini of Kv1.5 did confer PMA sensitivity to mature Kv1.4 channels, suggesting the involvement of Kv1.5 C-terminus in the channel ubiquitination. Removal of each of the potential ubiquitination residue Lysine at position 536, 565, and 591 by Arginine substitution (K536R, K565R, and K591R) had little effect, but removal of all three Lysine residues with Arginine substitution (3K-R) partially reduced PMA-mediated Kv1.5 degradation. Furthermore, removing the cysteine residue at position 604 by Serine substitution (C604S) drastically reduced PMA-induced channel degradation. Removal of the three Lysines and Cys604 with a quadruple mutation (3K-R/C604S) or a truncation mutation (Δ536) completely abolished the PKC activation-mediated degradation of Kv1.5 channels. These results provide mechanistic insight into PKC activation-mediated Kv1.5 degradation.

The voltage-gated potassium (Kv) channel, Kv1.5, encoded by the *KCNA5* gene on chromosome 12 belongs to the Shaker-related superfamily of Kv channels (1). Like other Kv channels, a single Kv1.5 channel is a tetramer assembled from four monomers or subunits. Each subunit consists of six transmembrane segments S1 to S6, and cytosolic N- and C- termini. The loop between S5 and S6 contributes to the permeation pore of the channel. Kv1.5 is expressed in atrial myocytes (2, 3), pancreatic beta cells (4), and smooth muscle cells (5, 6). In the heart, Kv1.5 is specifically expressed in the atria and conducts the ultra-rapidly activating delayed rectifier K^+^ current (I_Kur_) which contributes to the repolarization of atrial action potentials (2, 3, 7). Dysfunction of Kv1.5 due to acquired (8) or hereditary causes (9, 10) can lead to cardiac arrhythmias such as atrial fibrillation. As well, pharmacological targeting of Kv1.5 channels represents a means for the treatment of atrial fibrillation (11, 12, 13).

Protein kinase C (PKC) is a family of serine/threonine protein kinases that plays a central role in regulating biological functions under physiological and pathophysiological conditions. PKC activation is associated with alpha-adrenergic receptor stimulation and is prevalent in cardiovascular diseases like heart failure and cardiac hypertrophy (14, 15, 16, 17, 18). Activation of PKC regulates the Kv1.5 channel (19, 20), but the molecular mechanisms still need to be elucidated. Our previous study revealed that activation of PKC by phorbol 12-myristate 13-acetate (PMA) induces endocytic degradation of cell-surface Kv1.5 channels (21). Through truncation and deletion of N-terminal amino acid residues, we narrowed down the potential phosphorylation site to be the Threonine (Thr) residue at position 15 in the N-terminus of the channel (21). A point mutation removing the putative phosphorylation site, T15A, completely abolished the PMA effect. We thus proposed that PKC-mediated phosphorylation of Thr15 led to endocytic degradation of cell-surface Kv1.5 channels (21). However, molecular mechanisms underlying PMA-mediated Kv1.5 degradation are not well understood.

In the present study, we first demonstrated that PMA treatment enhanced the ubiquitination of Kv1.5 channels. By taking advantage of the Kv1.4 channel, another member of the Shaker Kv family, that is not affected by PKC activation, we made chimeric channels by swapping the N- and/or C-termini between Kv1.5 and Kv1.4 to determine the region of ubiquitination. We then made various mutations in Kv1.5 to investigate the ubiquitination sites involved in PKC-mediated channel degradation. Our results identified the specific ubiquitination sites in the C-terminus of Kv1.5 that are involved in PKC activation-mediated channel degradation.

## Results

### An intact intracellular environment is required for PMA treatment-induced reduction of I_Kv1.5_

**Figure 1:**
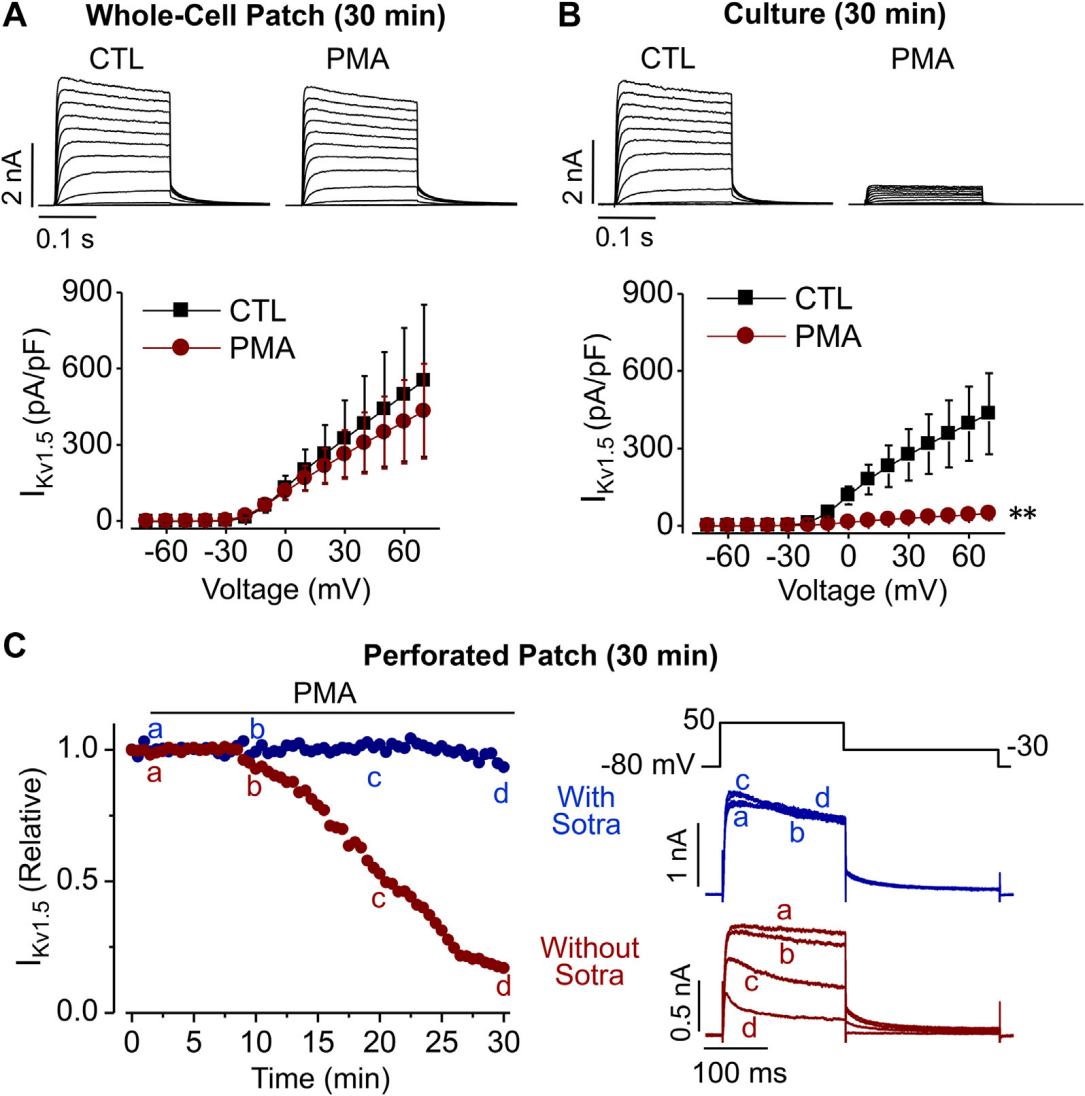
**Intact intracellular environment is required for PMA-induced reduction in I**_**Kv1.5**_**.***A*, PMA (10 nM, 30 min) application had no effect on I_Kv1.5_ during the whole-cell patch clamp configuration. Representative current traces are shown above summarized current-voltage relationships (n = 6 cells for each group from 3 independent experiments). *B*, PMA (10 nM, 30 min) application to intact cells reduced I_Kv1.5_. Representative current traces are shown above summarized current-voltage relationships (n = 15 cells for control (CTL), n = 18 cells for PMA treatment from four independent experiments). ∗∗*p* < 0.01 *versus* CTL for current amplitudes upon ≥10 mV depolarizing steps. *C*, relative I_Kv1.5_ continuously recorded with the voltage protocol shown above the current traces every 30 s using perforated-patch whole-cell clamp from cells exposed to PMA (10 nM) in the absence (*red*) or presence (*blue*) of the PKC inhibitor sotrastaurin (Sotra, 200 nM). Current traces at time points of ‘a’, ‘b’, ‘c’, and ‘d’ are superimposed for each condition.

The phorbol ester PMA is a direct activator of PKC, and we previously demonstrated that activation of PKC by 10 nM PMA decreased Kv1.5 currents (I_Kv1.5_) in HEK 293 cells stably expressing Kv1.5 channels (Kv1.5-HEK cells); and the PMA-mediated decrease in I_Kv1.5_ was completely prevented by treating cells with various PKC inhibitors including bisindolylmaleimide I, sotrastaurin, or staurosporine (21). To confirm that PMA does not directly affect I_Kv1.5_, we first achieved whole-cell patch clamp configuration of Kv1.5-HEK cells. After recording I_K1.5_ under the normal bath solution as control, 10 nM PMA was applied to the bath solution and I_Kv1.5_ was recorded for 30 min in the presence of PMA. PMA did not affect I_Kv1.5_ under this condition (Fig. 1*A*). In contrast, when 10 nM PMA was added to the medium during cell culture for 30 min, and cells were subsequently examined using whole-cell patch clamp recordings, I_Kv1.5_ was largely reduced compared to control cells (0.01% DMSO, vehicle for PMA) (Fig. 1*B*). The intracellular environment, thus the intact signaling for protein trafficking is usually disrupted by pipette solution under whole-cell patch clamp configuration (22). To directly observe the PMA-induced reduction in I_Kv1.5_ in cells with an intact intracellular environment, we performed perforated-patch whole-cell recordings using the pore-forming antibiotic nystatin (22). I_Kv1.5_ was elicited with the depolarizing step to 50 mV from the holding potential of −80 mV every 30 s for 30 min. As shown in Figure 1*C*, PMA (10 nM) induced a slowly developed progressive reduction of I_Kv1.5_, which was prevented by PKC inhibitor sotrastaurin (Sotra, 200 nM). These results indicate that PMA does not directly affect I_Kv1.5_ but does decrease I_Kv1.5_ through PKC activation-mediated loss of channel function, and consequently, endocytic degradation of mature Kv1.5 channels as we previously reported (21).

### PKC activation by PMA leads to ubiquitin (Ub)-mediated degradation of Kv1.5

**Figure 2:**
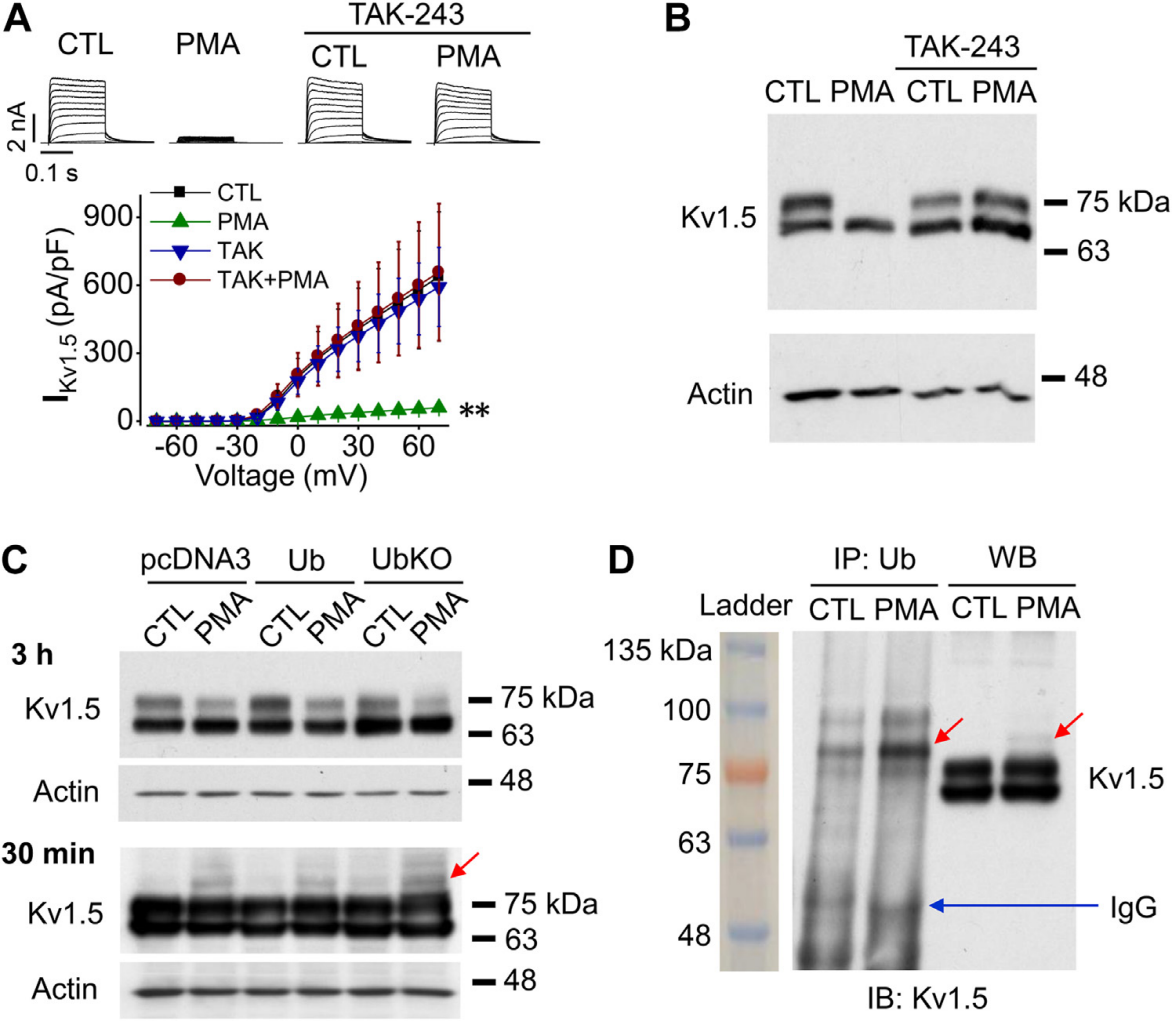
**Ubiquitination is involved in PMA-induced decrease in I**_**Kv1.5**_**and mature channel expression.***A*, inhibition of ubiquitination by 5 μM TAK-243 (TAK) abolishes the PMA (10 nM, 3 h)-induced reduction in I_Kv1.5_. Representative current traces are shown above summarized current-voltage relationships. ∗∗*p* < 0.01 *versus* CTL for current amplitudes upon ≥10 mV depolarizing steps. *B*, inhibition of ubiquitination by 5 μM TAK-243 abolishes the PMA (10 nM, 3 h)-induced reduction in expression of mature Kv1.5 proteins. β-Actin (42 kDa) was used as a loading control (n = 4). *C*, effects of PMA treatment for 30 min or 3 h on the expression of Kv1.5 channels in Kv1.5-HEK cells transfected with empty vector (pcDNA3), WT Ub, or mutant Ub, UbKO. *D*, Co-immunoprecipitation analysis shows that ubiquitination of mature Kv1.5 channels is increased by PMA treatment (10 nM, 30 min) (n = 8). *Red arrows* indicate ubiquitinated Kv1.5 proteins. CTL, control.

Ubiquitination is a common post-translational modification that drives the degradation of plasma membrane proteins (23, 24). To investigate the role of ubiquitination in the PMA-induced reduction in Kv1.5 current and expression, we utilized TAK-243, a small molecule inhibitor of ubiquitin (Ub)-activating enzyme, which blocks Ub conjugation thus disrupting protein ubiquitination (25). Kv1.5-HEK cells were treated with 10 nM PMA for 3 h in the absence (control) or presence of TAK-243 (5 μM). Patch clamp recording showed PMA treatment for 3 h markedly reduced I_Kv1.5_, and TAK-243 completely prevented this PMA-induced reduction in I_Kv1.5_ (Fig. 2*A*). Western blot analysis was used to investigate the effects of TAK-243 on Kv1.5 expression levels. On Western blot analysis, Kv1.5 proteins displayed two bands at 75 kDa and 68 kDa. The 75-kDa band represents the fully glycosylated mature form of Kv1.5 localized in the plasma membrane, responsible for conducting Kv1.5 current (I_Kv1.5_); the 68-kDa band represents the immature core-glycosylated form of Kv1.5 in the endoplasmic reticulum (26, 27). Consistent with our previous publication (21), PMA (10 nM) treatment essentially abolished the 75-kDa Kv1.5 protein. This effect was completely prevented by TAK-243 (5 μM) (Fig. 2*B*). Thus, ubiquitination is involved in the PMA-induced reduction in I_Kv1.5_ and mature Kv1.5 protein expression.

For ubiquitination-mediated degradation of cell membrane proteins including Kv1.5 channels, Ub attaches to the target protein and triggers protein internalization and degradation (28, 29). To assess if Kv1.5 channels go through Ub modification upon PKC activation, Kv1.5-HEK cells were treated with PMA (10 nM) for 30 min or 3 h. A 30-min treatment largely reduced I_Kv1.5_ (Fig. 1, *B* and *C*) but did not affect Kv1.5 protein expression level (Fig. 2*C*) (21) although a 3 h treatment caused a marked reduction of the mature (75-kDa) protein expression (Fig. 2, *B* and *C*) (21). As ubiquitination and its mediated degradation occur concurrently, the fraction of ubiquitinated channels at a given time point would be small. In order to detect ubiquitinated Kv1.5 channel proteins, the membrane-film was overexposed to focus on ubiquitinated proteins. Western blot analysis of Kv1.5 protein expression revealed that PMA treatment for 30 min induced an additional band at 83-kDa (Fig. 2*C*). As the molecular mass of a single Ub is 8.6 kDa, the 83-kDa band is most likely to be the monoubiquitinated mature (75 kDa) Kv1.5 channels. Monoubiquitination is known to trigger internalization and degradation of various channels (30, 31, 32). To further examine the ubiquitination of Kv1.5 channels upon PMA treatment, we transfected Kv1.5-HEK cells with wild-type (WT) Ub as well as a mutant Ub, UbKO, in which all seven lysine residues are replaced by arginine, and thus preventing the formation of polyubiquitin chains but allowing monoubiquitination (31, 32). Twenty-four hours after transfection, these cells were treated with 10 nM PMA for 30 min. Similar to pcDNA3-transfected cells, the potentially ubiquitinated Kv1.5 with an 83-kDa band was also observed in either Ub-transfected or UbKO-transfected cells (Fig. 2*C*, red arrow). Next, we performed co-immunoprecipitation experiments on whole-cell lysates from Kv1.5-HEK cells treated without (control, CTL) or with 10 nM PMA for 30 min. Proteins were precipitated using an anti-ubiquitin (Ub) antibody and probed with an anti-Kv1.5 antibody. In Ub-precipitated proteins, Kv1.5 expression with molecular masses of 83 (red arrows) as well as 92 kDa were observed which was enhanced by PMA treatment, indicating that PKC activation induces monoubiquitination as well as multi-monoubiquitination of Kv1.5 mature channels (Fig. 2*D*). These results indicate that PKC activation promotes the monoubiquitination of Kv1.5 channels, which triggers the reduction in I_Kv1.5_ as well as mature Kv1.5 expression.

### Ubiquitination is responsible for internalization as well as degradation of cell-surface Kv1.5 channels *via* the lysosomal pathway

**Figure 3:**
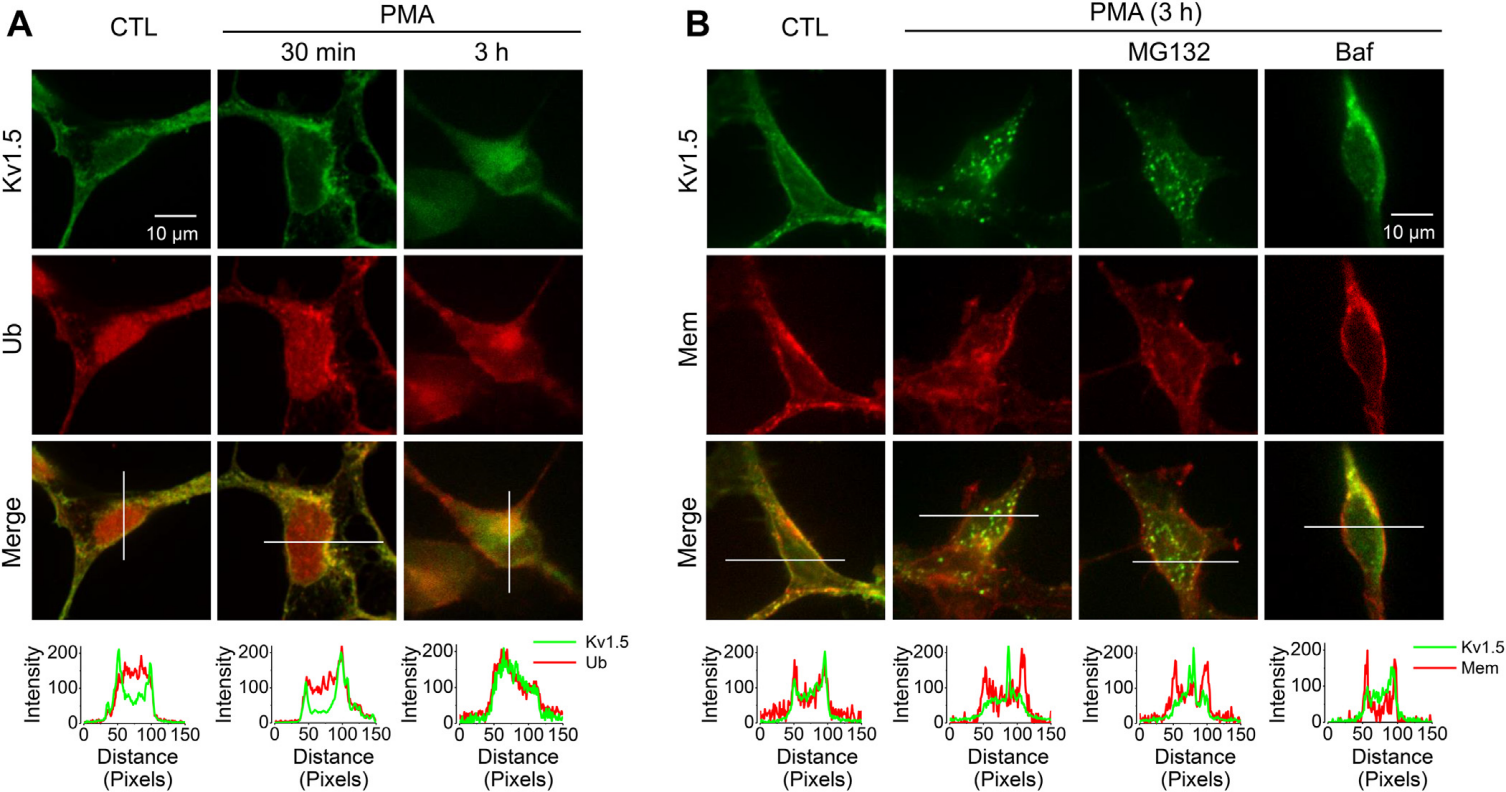
**PMA treatment induces Kv1.5 internalization involving Ub *via* lysosomal degradation.***A*, confocal images portraying the localization of Kv1.5 (*green*) and Ub (*red*) in Kv1.5-HEK cells in control (CTL) as well as in PMA treatment (10 nM) for 30 min or 3 h. *B*, confocal images portraying the localization of Kv1.5 (*green*) in cells in control (CTL) and in PMA treatment (10 nM, 3 h) in the absence or presence of proteasomal inhibitor MG132 (10 μM) or lysosomal inhibitor bafilomycin A1 (Baf, 1 μM). Cell membranes (*Mem*) were labeled with *Texas Red* X-conjugated wheat germ agglutinin (*red*). The signal intensity distributions along the white lines were analyzed using ImageJ.

To determine the interactions between Kv1.5 and Ub during the endocytic route of Kv1.5 internalization, we examined the colocalization between Kv1.5 and Ub in Kv1.5-HEK cells. Cells in control as well as cells with PMA (10 nM) treatment for 30 min or 3 h were fixed and permeabilized. Ub and Kv1.5 were labeled with appropriate primary and secondary antibodies. As shown in Figure 3*A*, under control conditions, Kv1.5 channels (green) were localized throughout the cell and concentrated in the plasma membrane. Ub (red) was evenly distributed throughout the cell. After a 30-min treatment with PMA, Kv1.5 channels were clustered in the membrane with some punctate staining, which suggests that Kv1.5 channels aggregated prior to internalization. Interestingly, Ub displayed a similar pattern of cellular distribution and colocalized with Kv1.5 channels. After a 3-h treatment with PMA, considerable Kv1.5 channels were internalized, and Ub displayed the same pattern of intracellular localizations and was colocalized with Kv1.5 channels. These results indicate that upon PMA treatment, ubiquitination of Kv1.5 channels triggers channel internalization as well as degradation.

Monoubiquitination is usually associated with the endocytosis and lysosomal sorting of plasma membrane proteins, whereas the formation of lysine-linked polyubiquitin chains is the primary signal for modified proteins to the 26S proteasome for degradation (33). As PMA induces monoubiquitination of Kv1.5 channels (Fig. 2), it is likely that PMA-induced reduction of Kv1.5 is through lysosomal degradation. To test this notion, the proteasomal inhibitor MG132 (10 μM) or the lysosomal inhibitor bafilomycin A1 (Baf, 1 μM) was included during PMA (10 nM) treatment of Kv1.5-HEK cells for 3 h. Cells in control as well as cells with PMA treatment were then fixed and permeabilized. Anti-Kv1.5 primary and secondary antibodies were used to stain Kv1.5 (green), whereas Texas Red-X conjugated wheat germ agglutinin was used to label the membrane. As shown in Figure 3*B*, Baf, but not MG132, prevented PMA-induced internalization of Kv1.5 channels. These data indicate PKC activation promotes monoubiquitination of cell-surface Kv1.5 channels and their degradation through the lysosomal pathway.

### Characterizations of chimeric Kv1.4/1.5 channels reveal the role of the Kv1.5 C-terminus in PMA-induced channel degradation

**Figure 4:**
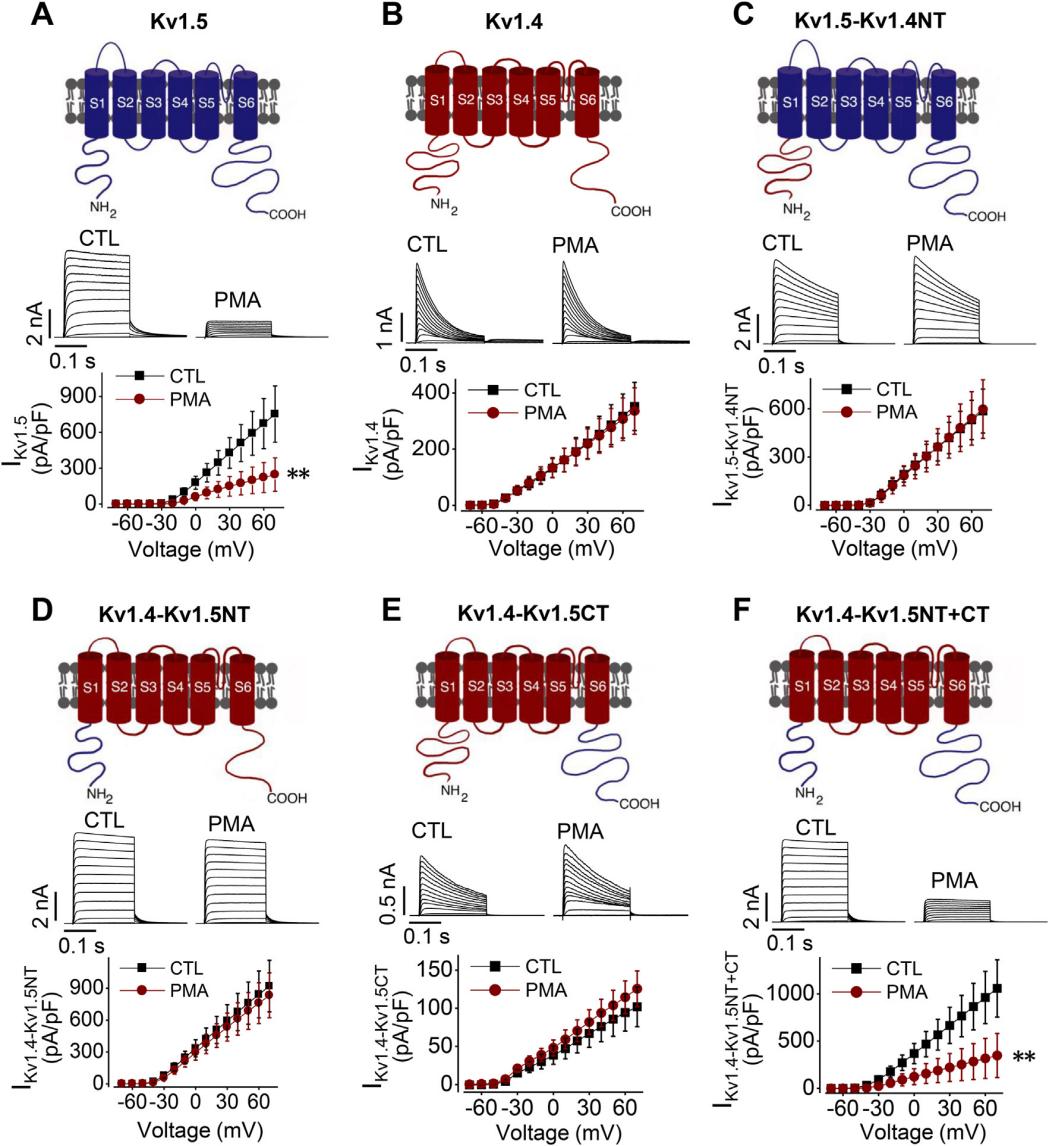
**Both N- and C-termini of Kv1.5 are involved in PKC activation-mediated channel degradation.***Upper panels*: Schematic diagrams of WT Kv1.5 (*A*), WT Kv1.4 (*B*), chimeric Kv1.5 with Kv1.4 N-terminus, Kv1.5-Kv1.4NT (*C*), chimeric Kv1.4 with Kv1.5 N-terminus, Kv1.4-Kv1.5NT (*D*), chimeric Kv1.4 with Kv1.5 C-terminus, Kv1.4-Kv1.5CT (*E*), or chimeric Kv1.4 with both Kv1.5 N- and C-termini, Kv1.4-Kv1.5NT+CT (*F*). *Middle and lower panels*: Representative current traces and summarized current-voltage relationships showing the effects of 10 nM PMA treatment for 3 h on the channels illustrated in the *upper panels* (n = 13–27 cells from 3 independent experiments for each group). ∗∗*p* < 0.01 *versus* control (CTL) for current amplitudes upon ≥10 mV depolarizing steps. Data are presented as mean ± SD.

As we have reported before, although the Kv1.4 channel also belongs to the Shaker superfamily of potassium channels, it is not susceptible to PMA-mediated degradation (21) (Fig. 4). Previously, we demonstrated that the Threonine residue at position 15 (Thr15) in the N-terminus of the channel is important for PKC activation-mediated reduction in Kv1.5 current and expression (21). A point mutation (T15A), deletion mutation (Δ2-19), or truncation mutation (ΔN209) that disrupts Thr15 abolishes Kv1.5 sensitivity to PMA treatment (21). Kv1.4 lacks the equivalent Thr15 residue in its N-terminus. We therefore swapped N-termini between Kv1.4 and Kv1.5 to make chimeric channels which were then expressed in HEK293 cells. As illustrated in Figure 4, treatment of cells with 10 nM PMA for 3 h decreased the current of Kv1.5 channel (Fig. 4*A*) but not the Kv1.4 channel (Fig. 4*B*). The PMA treatment did not affect the current of chimeric Kv1.5 with Kv1.4 N-terminus (Kv1.5-Kv1.4NT) (Fig. 4*C*), consistent with our previous observation that Thr15 in N-terminus plays a critical role (21). However, PMA treatment also did not affect the current of the chimeric Kv1.4 with Kv1.5 N-terminus (Kv1.4-Kv1.5NT, Fig. 4*D*), indicating that the presence of the Kv1.5 N-terminus alone is not sufficient to confer PKC sensitivity, despite containing the Thr15 residue. To investigate the role of the C-terminus of Kv1.5 in PMA-induced reduction in I_Kv1.5_, we replaced the C-terminus of Kv1.4 with that of Kv1.5 (Kv1.4-Kv1.5CT). PMA treatment did not affect the current of the chimeric Kv1.4-Kv1.5CT (Fig. 4*E*). Interestingly, when both the N- and C- termini of Kv1.4 were replaced with those of Kv1.5 (Kv1.4-Kv1.5NT+CT), PMA treatment led to a decrease in the current of Kv1.4-Kv1.5NT+CT channels similar to that seen in WT-Kv1.5 channels (Fig. 4*F*). These findings indicate the critical role of C-terminus in PKC-mediated Kv1.5 reduction.

### Lysine residues in the C-terminus are involved in PKC-mediated Kv1.5 reduction

**Figure 5:**
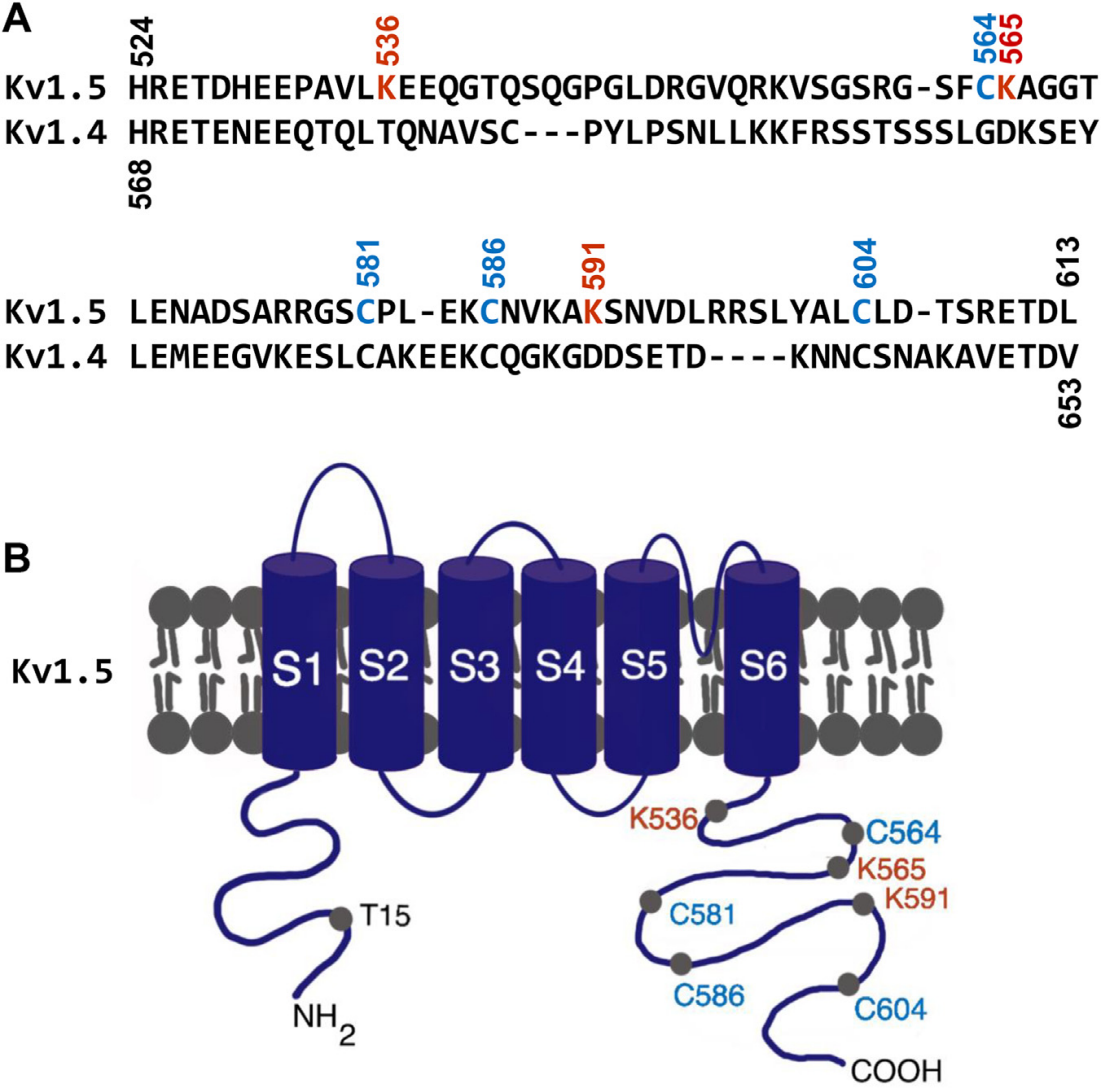
**Location of Cysteine and Lysine residues on the C-terminus of Kv1.5.***A*, sequence alignments of the C-terminus of Kv1.4 and Kv1.5 with cysteine and lysine residues investigated in the present study as potential ubiquitination sites. *B*, illustrative locations of the investigated Lysine and Cysteine residues in Kv1.5 C-terminus.

We previously proposed that PKC-mediated phosphorylation of Thr15 plays a role in PMA-induced Kv1.5 degradation from cell surface (21). The present study demonstrated that channel ubiquitination was also essential to PMA-induced Kv1.5 channel degradation (Fig. 2). Since replacing the N-terminus alone did not, but replacing both the N- and C-termini of Kv1.4 with the N- and C-termini of Kv1.5 did confer the PMA-induced channel degradation, the ubiquitination sites are most likely to be in the C-terminus of Kv1.5. Ubiquitination is a common signal for endocytic degradation of membrane proteins including ion channels (23), and Ub most commonly acts on lysines (Lys), and also on cysteines (Cys) (34). Sequence analyses of the C-termini between Kv1.5 and Kv1.4 identified three Lys residues unique to the Kv1.5 C-terminus as possible candidates for ubiquitination sites, Lys536, Lys565, and Lys591 (Fig. 5, *A* and *B*, red residues).

**Figure 6:**
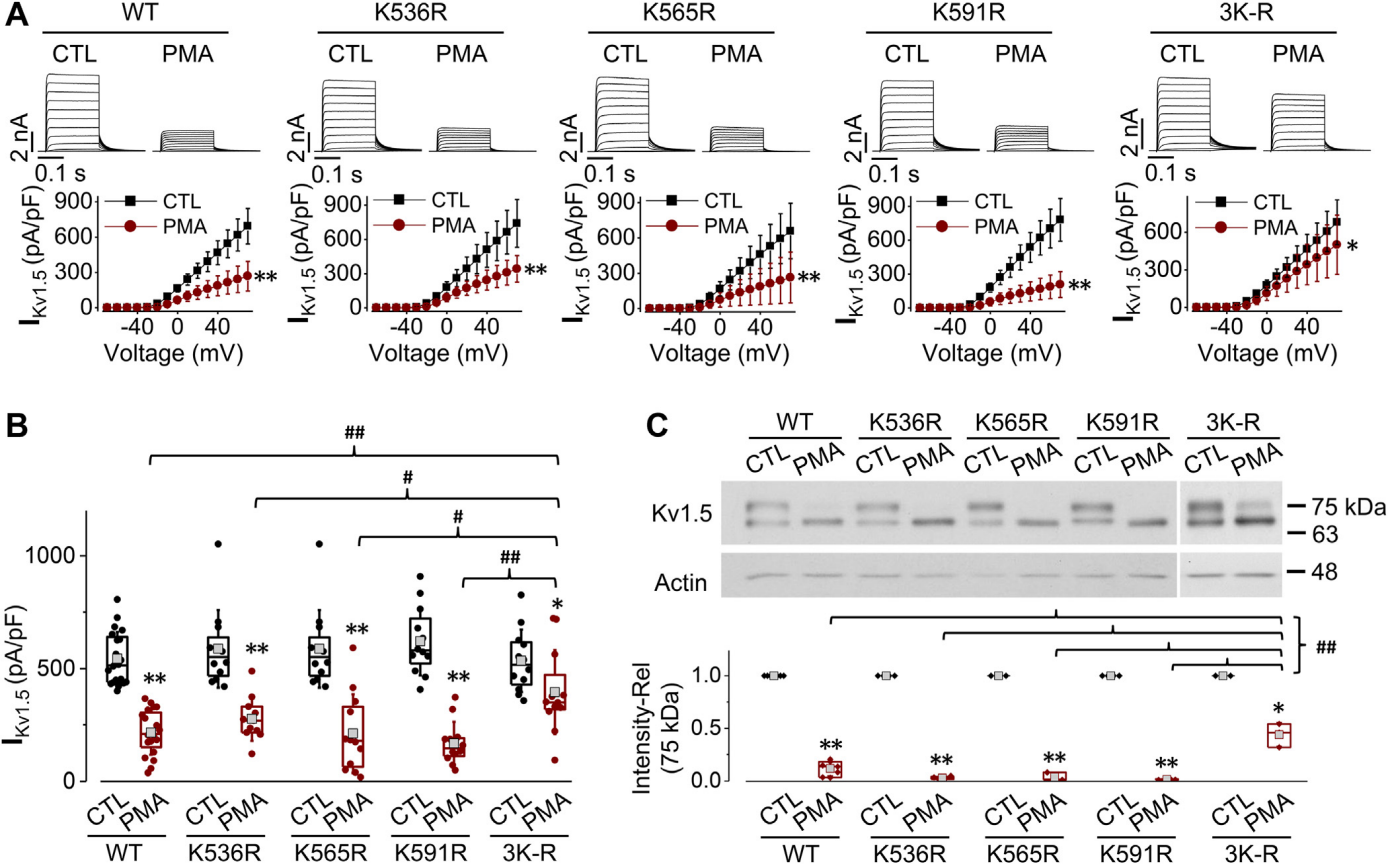
**Role of lysine residues on the C-terminus of Kv1.5 in PMA-induced reduction in I**_**K1.5**_**and mature Kv1.5 channel expression.***A*, the effects of PMA treatment (10 nM, 3 h) on I_Kv1.5_ of WT Kv1.5, Kv1.5-K536R, Kv1.5-K565R, Kv1.5-K591R, and Kv1.5-3K-R (triple lysines changed to arginines) mutants. Representative current traces and summarized current-voltage relationships are shown. ∗*p* < 0.05, ∗∗*p* < 0.01 *versus* control (CTL) for current amplitudes upon ≥10 mV depolarizing steps. *B*, peak I_Kv1.5_ density is shown as box plots with mean ± SD in control (CTL) and in PMA treatment (10 nM, 3 h) for WT and mutant channels. The PMA-induced reduction in I_Kv1.5_ of the triple mutant is significantly weakened than that seen in WT Kv1.5 or in single-point mutants. ∗*p* < 0.05, ∗∗*p* < 0.01 *versus* CTL; ^#^*p* < 0.05, ^##^*p* < 0.01 *versus* indicated groups. *C*, the effects of PMA treatment (10 nM, 3 h) on the expression of WT Kv1.5, Kv1.5-K536R, Kv1.5-K565R, Kv1.5-K591R, and Kv1.5-3K-R mutants. The intensity of the 75-kDa band in each lane was firstly normalized to the loading control actin (42-kDa) in the same lane, then normalized to its control, and summarized as box plots with mean ± SD beneath the representative Western blot images. n = 3 to 8, ∗*p* < 0.05, ∗∗*p* < 0.01 *versus* CTL; ^##^*p* < 0.01 *versus* indicated groups.

To determine the role of these lysine residues in the C-terminus of Kv1.5, point mutations were created where arginine was used to replace the lysine as they both carry the positive charge, thus, such a replacement may not markedly affect protein folding but impair Ub binding. Mutant Kv1.5 cDNA plasmids were created and transfected into HEK293 cells to establish stable cell lines expressing Kv1.5-K536R, Kv1.5-K565R, and Kv1.5-K591R mutants, respectively. These cells were then treated with 10 nM PMA for 3 h and analyzed using whole-cell voltage clamp recordings and Western blot analysis. PMA treatment decreased the currents of Kv1.5-K536R, Kv1.5-K565R, and Kv1.5-K591R to an extent comparable to that of WT Kv1.5 (Fig. 6, *A* and *B*). Consistently, Western blots showed that PMA treatment drastically decreased the mature Kv1.5 protein expression (upper band) of WT channels as well as these three mutant channels (Fig. 6*C*).

Ubiquitination can occur at multiple sites, so the removal of one single site may not be enough to affect PMA-induced endocytic degradation as the ubiquitin may bind to alternate sites (35, 36). We thus created a triple mutant that changed all three lysines to arginines (Kv1.5-K536R/K565R/K591R, Kv1.5-3K-R) and made a HEK293 cell line stably expressing the Kv1.5-3K-R, which was treated with 10 nM PMA for 3 h. Compared to WT Kv1.5 or any of the single-site mutants, the triple lysine-to-arginine mutant Kv1.5 (Kv1.5-3K-R) attenuated the PMA-induced reduction in I_Kv1.5_ and mature protein expression level (Fig. 6, *A*–*C*). Nonetheless, Kv1.5-3K-R mutation did not completely abolish PMA-induced reduction in I_Kv1.5_ and mature protein expression, suggesting the existence of additional ubiquitin-binding site(s).

### Cysteine 604 in Kv1.5 C-terminus is also involved in ubiquitination and subsequent channel degradation

**Figure 7:**
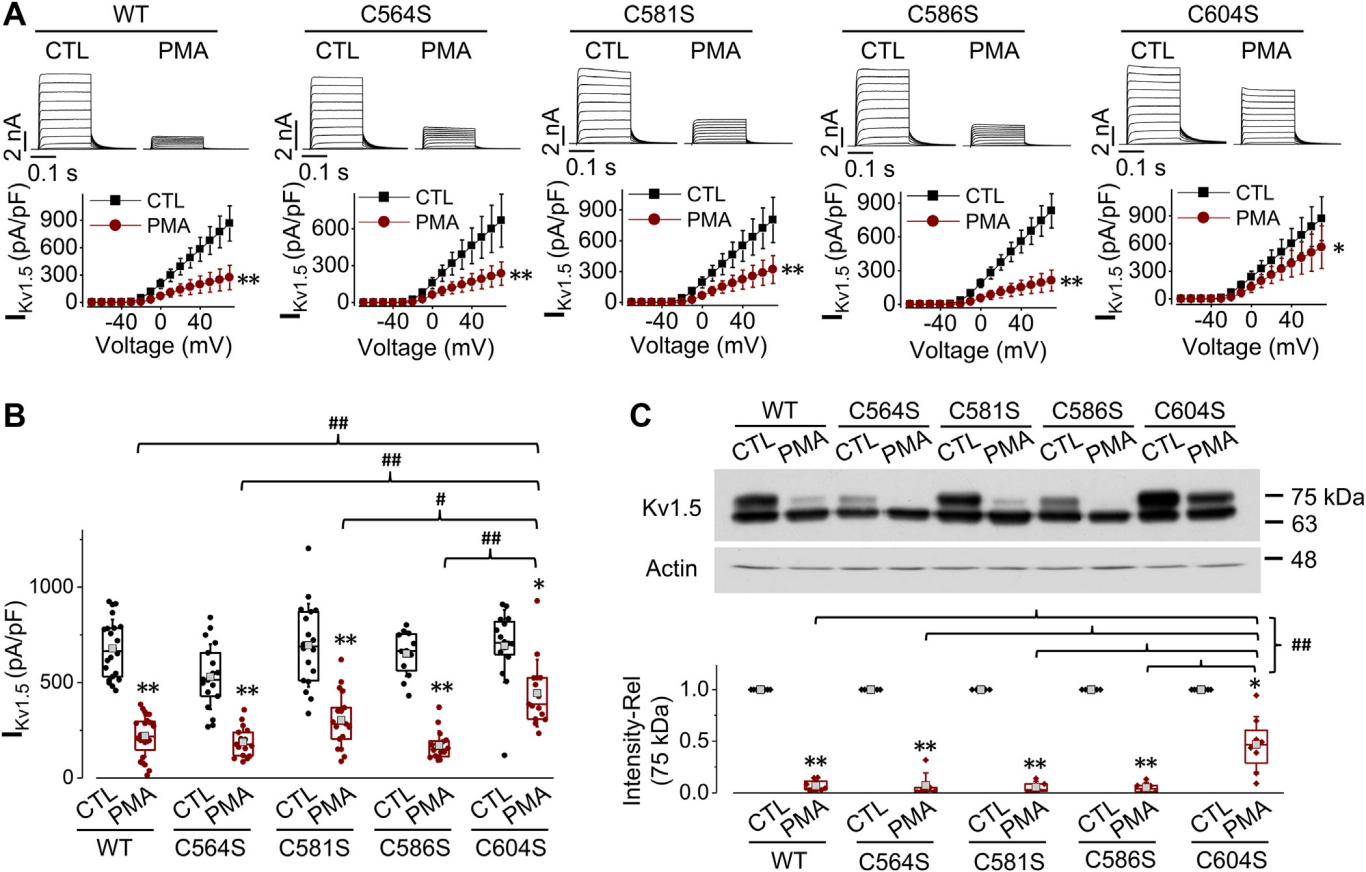
**Role of cysteine residues on the C-terminus of Kv1.5 in PMA-induced reduction in I**_**K1.5**_**and mature Kv1.5 channel expression.***A*, the effects of PMA treatment (10 nM, 3 h) on I_Kv1.5_ of WT Kv1.5, Kv1.5-C564S, Kv1.5-C581S, Kv1.5-C586S, and Kv1.5-C604S mutants. Representative current traces and summarized current-voltage relationships are shown. ∗*p* < 0.05, ∗∗*p* < 0.01 *versus* control (CTL) for current amplitudes upon ≥10 mV depolarizing steps. *B*, I_Kv1.5_ density is shown as box plots with mean ± SD in control (CTL) and in PMA treatment (10 nM, 3 h) for WT and mutant channels. The PMA-induced reduction in I_Kv1.5_ of Kv1.5-C604S is significantly weakened than that seen in WT Kv1.5 or in other mutant channels. ∗*p* < 0.05, ∗∗*p* < 0.01 *versus* CTL; ^#^*p* < 0.05, ^##^*p* < 0.01 *versus* indicated groups. *C*, the effects of PMA treatment (10 nM, 3 h) on the expression of WT Kv1.5, Kv1.5-C564S, Kv1.5-C581S, Kv1.5-C586S, and Kv1.5-C604S mutants. The intensity of the 75-kDa band in each lane was firstly normalized to the loading control actin (42-kDa) in the same lane, then normalized to its control, and summarized as box plots with mean ± SD beneath the representative Western blot images. n = 5 to 8, ∗*p* < 0.05, ∗∗*p* < 0.01 *versus* CTL; ^##^*p* < 0.01 *versus* indicated groups.

Evidence shows that ubiquitination can also occur in the sulfhydryl group (37, 38, 39). Thus, we investigated the potential role of cysteine residues in the C-terminus of Kv1.5 involved in PMA-induced channel degradation. Four cysteine residues of interest were identified: Cys564, Cys581, Cys586, and Cys604 (Fig. 5, *A* and *B*, blue residues). Point mutation with Kv1.5-C564S, Kv1.5-C581S, Kv1.5-C586S, or Kv1.5-C604S was created where the cysteine was replaced with serine, as serine is structurally similar to cysteine but lacks the sulfhydryl group. HEK293 cell lines stably expressing Kv1.5-C564S, Kv1.5-C581S, Kv1.5-C586S, or Kv1.5-C604S respectively were created and treated with 10 nM PMA for 3 h. Although PMA caused a reduction in I_Kv1.5_ and mature channel (75-kDa) expression in all the cysteine mutated channels, the Kv1.5-C604S displayed a much-reduced sensitivity compared to WT and the other mutants Kv1.5-C564S, Kv1.5-C581S, or Kv1.5-C586S (Fig. 7, *A*–*C*). These results indicate that Cys604 may be an important site for PMA-induced Kv1.5 channel degradation.

### Combined C604S and triple lysine mutation as well as C-terminus truncation at residue 536 completely abolish PMA-induced Kv1.5 channels degradation

**Figure 8:**
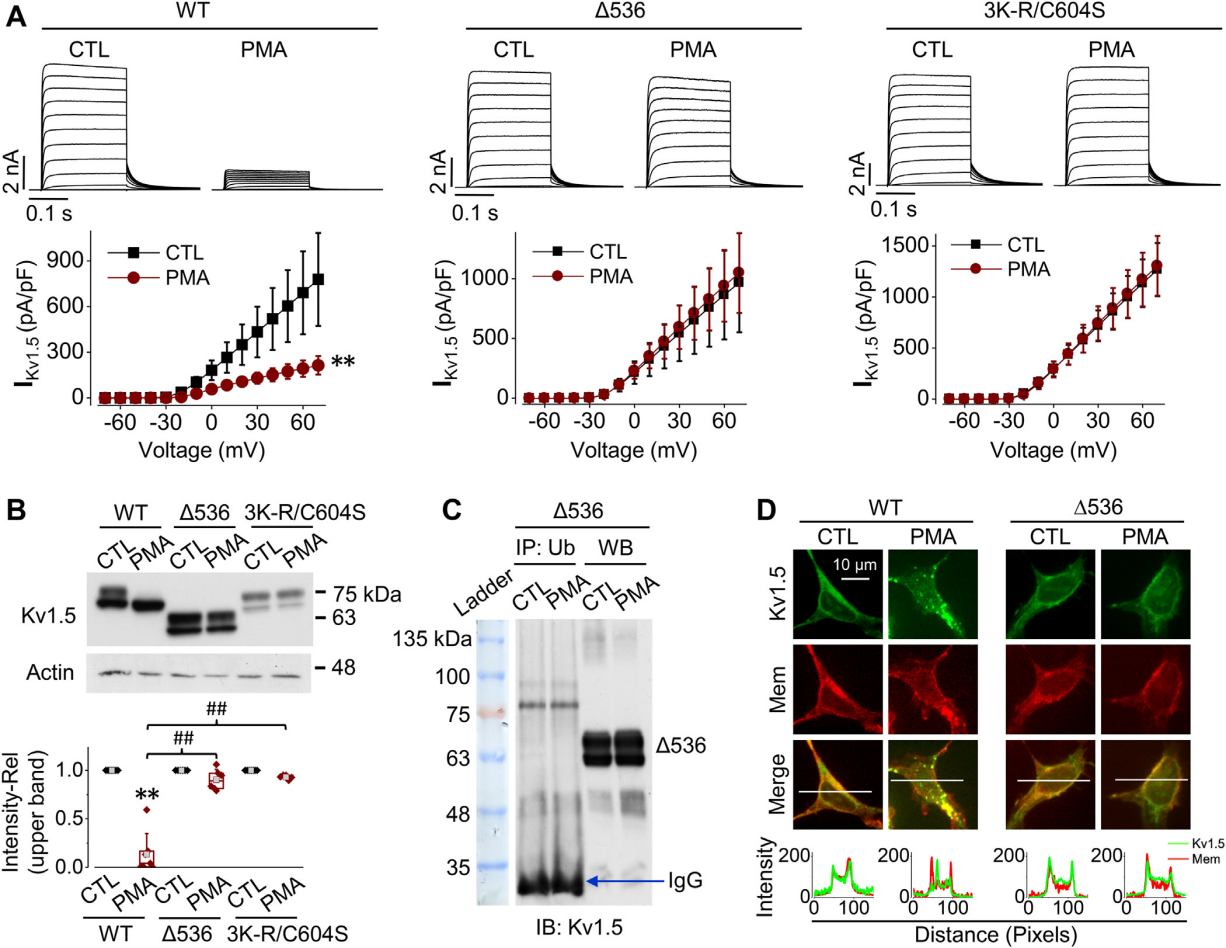
**Removing all potential ubiquitination sites completely abolishes PMA-induced reduction in I**_**K1.5**_**and mature Kv1.5 channel expression.***A*, the effects of PMA treatment (10 nM, 3 h) on I_Kv1.5_ of WT Kv1.5, Kv1.5-Δ536 and Kv1.5-3K-R/C604S. Representative current traces and summarized current-voltage relationships are shown. n = 10 to 12 cells for WT Kv1.5, n = 17 to 18 cells for Kv1.5-Δ536, n = 17 to 18 cells for Kv1.5-3K-R/C604S. For each channel, at least 3 independent experiments were performed. ∗∗*p* < 0.01 *versus* control (CTL). *B*, the effects of PMA treatment (10 nM, 3 h) on the expression of WT Kv1.5, Kv1.5-Δ536, and Kv1.5-3K-R/C604S channels. The intensity of the upper band in each lane was firstly normalized to the loading control actin (42-kDa) in the same lane, then normalized to its control, and summarized as box plots with mean ± SD beneath the representative Western blot images. n = 5 to 7, ∗∗*p* < 0.01 *versus* CTL; ^##^*p* < 0.01 *versus* WT with PMA. *C*, co-immunoprecipitation analysis shows that PMA treatment had no effect on the ubiquitination of Kv1.5-Δ536 channels (n = 4). *D*, immunofluorescence images portraying the localization of Kv1.5 in HEK293 cells stably expressing WT Kv1.5 or Kv1.5-Δ536 after culture with 10 nM PMA for 3 h. Kv1.5 was detected with anti-Kv1.5 primary antibody and Alexa Fluor 488-conjugated secondary antibody (*green*). Cell membranes (*Mem*) are labeled with Texas Red X-conjugated wheat germ agglutinin (*red*). The signal intensity distributions along the white lines were analyzed using ImageJ.

Since both the triple lysine mutation (Kv1.5-3K-R) and Kv1.5-C604S mutation showed a decreased PMA sensitivity, we created a quadruple mutant in which 3K-R was combined with C604S (Kv1.5-3K-R/C604S) to investigate if the lysine residues and Cys604 work synchronously to mediate the effect of PKC activation induced by PMA. Additionally, we created a C-terminus truncation mutant (Δ536) which removes all the cysteine and lysine residues that were identified as potential ubiquitination sites (Fig. 5). The quadruple mutant (3K-R/C604S) or C-terminal truncation mutant (Δ536) was transfected into HEK293 cells which were then treated with 10 nM PMA for 3 h. Whole-cell voltage-clamp data showed that both I_Kv1.5_ of Kv1.5-3K-R/C604S and Kv1.5-Δ536 mutants were completely unaffected by PMA treatment (Fig. 8*A*). Consistently, Western blot analyses showed that unlike WT Kv1.5, Kv1.5-3K-R/C604S and Kv1.5-Δ536 mutants displayed no change in the mature channel expression of Kv1.5 after PMA treatment (Fig. 8*B*). Co-IP experiments were performed to examine the effects of PMA treatment on the ubiquitination of Kv1.5-Δ536 mutant. PMA (10 nM, 30 min) treatment did not induce any ubiquitination of this mutant channel (Fig. 8*C*). The expression of Kv1.5 channels was also observed using immunocytochemical staining. Under control conditions, WT Kv1.5 and Kv1.5-Δ536 channels were expressed in the plasma membrane as well as inside the cells. After treatment with PMA (10 nM, 3 h), the plasma membrane expression of WT Kv1.5, but not Kv1.5-Δ536, was markedly decreased along with internalized punctate staining (Fig. 8*D*). These results indicate that three lysine residues at position 536, 565, and 591, as well as a cysteine residue at position 604, are all ubiquitination sites that collectively participate in PKC activation-mediated degradation of mature (cell surface) Kv1.5 channels.

## Discussion

In the atria, the Kv1.5 channel is important for the repolarization of atrial action potentials, and its dysfunction is associated with atrial arrhythmias such as atrial fibrillation (2, 3, 7, 8, 9, 10, 11, 12, 13). PKC activation is known to occur in various physiological and pathophysiological conditions, such as heart failure and cardiac hypertrophy (15, 16, 18, 40). Our finding that PKC activation downregulates Kv1.5 density in the plasma membrane provides a possible mechanistic link between various cardiovascular pathologies such as heart failure and the increased prevalence of atrial arrhythmias such as atrial fibrillation (40).

We previously demonstrated that Thr15 in the N-terminus of Kv1.5 is involved in the PKC activation-mediated endocytic degradation of cell-surface Kv1.5 channels (21). In the present study, we demonstrate that PMA application (10 nM, 30 min) during the conventional whole-cell voltage clamp recordings did not affect I_Kv1.5_. However, PMA application (10 nM, 30 min) to Kv1.5-HEK cells in culture or during perforated-patch whole-cell recording markedly reduced I_Kv1.5_ (Fig. 1). In whole-cell recordings, the pipette solution used approximately matches the physiological intracellular fluid in aspects of ionic composition but lacks complex intracellular molecules such as Ub and associated enzymes critical for membrane protein trafficking (22). The exchange of fluids during whole-cell patch clamp configuration between pipette solution and intracellular fluid inevitably disturbs the originally intact intracellular environment. In contrast, perforated-patch whole-cell recording allows electrical access through small pores in the membrane which are permeable to small monovalent ions but not permeable to larger divalent ions and molecules, thus the intracellular environment is largely preserved. Therefore, we conclude that intracellular signaling is involved in PKC activation-induced reduction in I_Kv1.5_. Moreover, while a 30-min PMA treatment eliminated I_Kv1.5_ (Fig. 1), a 3-h PMA treatment is required to observe the marked reduction of the mature (75-kDa) Kv1.5 channel proteins (Fig. 2*B*). We believe that PKC activation induced a modification of Kv1.5 channels which then causes a conformational change, leading to the loss of conductivity, and further triggering channel internalization and degradation. The evidence for PMA-induced Kv1.5 modification includes channel ubiquitination which is also a common pathway mediating protein degradation (23). The Ub-activating enzyme inhibitor TAK-243 completely prevented the 3-h PMA-induced reduction in I_Kv1.5_ (Fig. 2*A*) and the mature (75 kDa) channel expression (Fig. 2*B*). By examining Kv1.5 expression in cell lysate and proteins immunoprecipitated using an anti-Ub antibody in Kv1.5-HEK cells after a 30 min treatment, we demonstrated that PMA treatment increased ubiquitination of Kv1.5 channels (Fig. 2, *C* and *D*). Our immunofluorescence results further indicate that ubiquitination is involved both for Kv1.5 internalization and degradation *via* the lysosomal but not the proteasomal pathway (Fig. 3). This is consistent with our previously published data obtained with a patch-clamp method and Western blot analyses (21).

We have demonstrated that the Kv1.4 channel is insensitive to PMA treatment (21). We thus made Kv1.5/Kv1.4 chimeric channels to determine the ubiquitination sites of Kv1.5. Introducing the N-terminus alone did not but introducing both N- and C-termini of Kv1.5 into PMA-insensitive Kv1.4 did confer the PMA sensitivity to the Kv1.4 chimeric channel (Fig. 4). This indicates that while phosphorylation of Thr15 in the N-terminus of Kv1.5 is important, the ubiquitination involved in PMA-mediated internalization occurs in the C-terminus. By comparing amino acid residues between PMA-sensitive Kv1.5 and PMA-insensitive Kv1.4, we identified several potential ubiquitination sites in the C-terminus of Kv1.5 (Fig. 5). We then focused on identifying ubiquitination sites in the C-terminus of Kv1.5 channels by constructing various mutant channels to replace or remove the potential lysine or cysteine residues. Although mutations in Kv1.5 can lead to non-functional proteins (10), remarkably in our study, mutations with various residues, as well as a truncation, yielded fully functional mutant channels capable of conducting robust currents, allowing us to identify residues participating in PMA-induced channel degradation.

Our mutagenesis investigation of target lysine residues showed that individual lysine to arginine point mutations had limited effect on PMA-induced Kv1.5 reduction. However, a triple mutation replacing all three lysines with arginines (Kv1.5-3K-R) weakened PMA-induced reduction in I_Kv1.5_ and mature Kv1.5 expression (Fig. 6). Additionally, we also studied the role of cysteines in the C-terminus by creating cysteine to serine point mutations (Kv1.5-C564S, Kv1.5-C581S, Kv1.5-C586S, Kv1.5-C604S). We found that only the point mutation of Cys604 greatly alleviated PMA-induced reduction in I_Kv1.5_ and mature Kv1.5 expression (Fig. 7). Combining the Kv1.5-C604S point mutation with the triple lysine mutation completely abolished PKC-mediated reduction in I_Kv1.5_ and mature Kv1.5 expression (Fig. 8). Thus, removing an individual ubiquitination site through site-directed mutagenesis has limited impact due to the redundancy of other sites, necessitating mutation of multiple residues. These findings suggest the presence of multiple ubiquitination sites in the C-terminus of Kv1.5 channels. A similar finding was previously reported in Organic Anion Transporter-1 (OAT1); it was shown that three lysine residues played a synergistic role in PKC-mediated ubiquitination of OAT1 (41). Studies have also shown that among multiple ubiquitination sites, one may play a predominant role (36, 42, 43). In the present study, our data show that Cys604 apparently plays a predominant role as the mutating Cys604 alone provided an impact similar to mutating all three Lys536, Lys565, and Lys591 (Figs. 6 and 7). Likewise, when Cys604 was removed, the effect of PKC activation was not fully abolished due to the presence of the three lysine residues. A complete abolishment of the effect of PKC activation was seen in the quadruple mutant or Δ536 truncation mutant where all three lysines and Cys604 were removed (Fig. 8). PMA treatment also did not induce ubiquitination of the Kv1.5-Δ536 mutant channels (Fig. 8*C*). Overall, these data clearly demonstrated that ubiquitination is involved in PKC-mediated degradation of Kv1.5 channels.

In a previous study, our data suggested that phosphorylation of Thr15 in the N-terminus is required for PKC activation-mediated reduction in I_Kv1.5_. In the present study, we show ubiquitination at multiple sites in the C-terminus of Kv1.5 also plays a critical role in this regulation. Thus, the phosphorylation site in the N-terminus communicates with the ubiquitination sites in the C-terminus to regulate the expression of Kv1.5 channels in the plasma membrane. While this has not been documented in Kv1.5, there is a growing body of literature that illustrates phosphorylation as a versatile switch in ubiquitination pathways (43, 44, 45). Interactions between phosphorylation and ubiquitination for Kv1.5 regulation warrant future investigation.

In summary, our present study demonstrated the involvement of multiple ubiquitination sites in PKC activation-mediated degradation of Kv1.5 channels, which consequently affect the density of mature Kv1.5 channels in the plasma membrane. We further identified the contributions of each of the four ubiquitination sites in this regulatory process. Our findings raise the possibility that the multiple ubiquitination sites provide a means of fine-tuning Kv1.5 density in the plasma membrane under various physiological and pathophysiological conditions.

## Experimental procedures

### Molecular biology

WT Kv1.5 in pcDNA3 was obtained from Dr Michael Tamkun (Colorado State University). Kv1.4 cDNA in pcDNA3.1 was purchased from GenScript. Mutant channels were created using the polymerase chain reaction (PCR) method and confirmed by DNA sequencing (Azenta Life Sciences). Point mutations include Lys to Arg (K536R, K565R, K591R, 3K-R) and Cys to Ser (C564S, C581S, C586S, and C604S) as well as the quadruple mutant (3K-R/C604S). The mutant Δ536 was created by mutating Lys-536 to a stop codon to achieve a truncation of C-terminus from Lys536. Chimeric channels between Kv1.5 and Kv1.4 were created by PCR amplification followed by insertion *via* restriction enzymes. The N-terminus of Kv1.4 was inserted to replace the N-terminus of Kv1.5 *via* 5′ KpnI and 3′ PmlI sites to create Kv1.5-Kv1.4NT. The N-terminus of Kv1.5 was inserted to replace the N-terminus of Kv1.4 *via* 5′ BamHI and 3′ BspEI sites to create Kv1.4-Kv1.5NT. The C-terminus of Kv1.5 was inserted to replace the C-terminus of Kv1.4 or Kv1.4-Kv1.5NT *via* 5′ BstEII and 3' AvrII to create Kv1.4-Kv1.5CT or Kv1.4-Kv1.5NT+CT, respectively. All mutant/chimeric channels were confirmed by sequencing.

Lipofectamine 2000 (Thermo Fisher Scientific) was used to transfect WT Kv1.5, or respective mutant plasmids into Human Embryonic Kidney (HEK) 293 cells. Stable cell lines expressing the channel proteins were made using G418 selection and the cells were cultured in Minimal Essential Medium supplemented with 10% fetal bovine serum, 1 mM sodium pyruvate, and nonessential amino acids (Thermo Fisher Scientific).

### Cell treatment

Cells were split into control groups and treatment groups. In the treatment group, cells were treated with 10 nM PMA (Sigma-Aldrich) for 30 min or 3 h in culture media in order to activate PKC. 5 μM TAK-243 (Cayman Chemical) was used to inhibit ubiquitin-activating enzyme. The proteasomal inhibitor MG132 (10 μM) or the lysosomal inhibitor bafilomycin A1 (Baf, 1 μM) was added during PMA (10 nM) treatment of Kv1.5-HEK cells for 3 h to examine endocytosis of Kv1.5 channels and the associated pathways.

### Western blot analysis

Whole-cell lysates were collected from cultured cells for Western blot analysis (26). Cells were washed with ice-cold Phosphate Buffered Saline (PBS), collected, and centrifuged at 1000*g* for 4 min. Ice cold lysis buffer containing 1 mM phenylmethylsulfonyl fluoride (PMSF; Sigma-Aldrich, St Louis, MO) and 1× protease inhibitor cocktail (Sigma-Aldrich) were added to the cell pellets which were then lysed using high-frequency sonication. The lysates were centrifuged at 10,000*g* for 10 min and the supernatant containing the proteins was collected. Protein concentrations were determined using a detergent-compatible protein assay kit (Bio-Rad, Hercules, CA). Samples comprised of 15 μg of protein diluted with double-distilled water and Laemmli loading buffer containing 5% β-mercaptoethanol were boiled at 100°C for 5 min. Then, samples along with BLUeye Prestained Protein Ladder (Bio-Helix, Taiwan) were loaded and separated on 8% SDS-polyacrylamide gel, then transferred onto a polyvinylidene difluoride (PVDF) membrane (Bio-Rad). Afterward, the membrane was blocked with 5% nonfat skim milk in Tris-buffered saline with 0.1% Tween-20 for 1 h at room temperature to prevent nonspecific protein interactions. The membrane was probed with appropriate primary antibodies (in 5% nonfat milk) for 2 h, then washed three times with 0.1% Tween 20 containing Tris-buffered saline and incubated with horseradish peroxidase (HRP)-conjugated secondary antibodies for 1 h. An enhanced chemiluminescence (ECL) detection kit (GE Healthcare) was used to visualize protein bands on X-ray film (Fujifilm). These films were scanned onto a computer and analyzed using Bio-Rad ImageLab software for quantification to determine band intensities with each band of interest being normalized to its respective actin value and expressed relative to the control in the same gel.

For immunoprecipitation, 0.5 mg of total protein was incubated with anti-Ub antibody overnight at 4 °C before precipitation with protein A/G plus-agarose beads at 4 °C for 4 h. The beads were washed 3 times with ice-cold lysis buffer, resuspended in 2× Laemmli sample buffer, and boiled for 5 min. The samples were then centrifuged at 10,000*g* for 5 min. The supernatants were analyzed using Western blotting to detect Kv1.5 expression.

### Electrophysiological recordings

Both conventional and perforated-patch whole-cell recordings were used to record I_Kv1.5_ at room temperature (22 ± 1 °C). Cells were settled at the bottom of a 0.5 ml perfusion chamber in a bath solution containing 135 mM NaCl, 5 mM KCl, 1 mM MgCl_2_, 2 mM CaCl_2_, 10 mM glucose, and 10 mM HEPES (pH 7.4 with NaOH). Patch glass pipettes were pulled from thin-walled borosilicate glass (World Precision Instruments, Sarasota, FL) and polished with heat until they have an inner diameter of 1.5 μm and a resistance of 2 MΩ. For conventional whole-cell clamp recordings, the pipette solution contained 135 mM KCl, 5 mM MgATP, 5 mM EGTA, and 10 mM HEPES (pH 7.2 with KOH). For perforated-patch whole-cell recording, nystatin was used as the pore-forming agent (22). The pipette solution consisted of 140 mM K-aspartate, 10 mM NaCl, 1 mM CaCl_2_, 1 mM MgCl_2,_ and 10 mM HEPES (pH 7.2 with KOH) (46). Nystatin was dissolved in DMSO as a stock solution (5 mg/100 μl) daily in dark, and was diluted in a pipette solution with a final concentration of 250 μg/ml. The nystatin-containing pipette solution was vortexed, sonicated in the water bath for about 30 s, and used within 2 h. To ease giga-ohm seals, the tip of the perforated-patch pipette was filled with nystatin-free solution. Electrical access through perforated patch membrane is indicated by capacitance currents. An Axopatch 200B amplifier and pCLAMP10 (Molecular Devices, San Jose, CA) were used for data acquisition and analysis. Data were sampled at 20 kHz and filtered at 5 kHz. For the time-dependent effects of PMA, I_Kv1.5_ was elicited with the depolarizing step to 50 mV from the holding potential of −80 mV every 30 s for 30 min. For recording a family of I_Kv1.5_, currents were elicited from the holding potential of −80 mV by depolarizing steps to voltages between −70 and +70 mV in 10 mV increments for 200 ms, followed by a repolarizing step to −30 mV for 250 ms before returning to the holding potential. Current-voltage (I-V) relationships were constructed by plotting the maximal currents during depolarizing steps against depolarizing voltages in each cell, data from multiple cells from at least 3 independent experiments were summarized. For box plots of I_Kv1.5_ density, currents upon 50 mV depolarizing steps were used.

### Immunofluorescence microscopy

HEK293 cells stably expressing WT Kv1.5 or Kv1.5-Δ536 mutant grown on glass coverslips were treated without (control) or with 10 nM PMA for 30 min, or 3 h alone or along with MG132 (10 μM) or Baf (1 μM). The membrane was stained with Texas Red X-conjugated wheat germ agglutinin (WGA, 5 μg/ml; Invitrogen) for 1 min at room temperature prior to cell fixation. Cells were washed with PBS and fixed using 4% ice-cold paraformaldehyde in PBS for 15 min, permeabilized with 0.1% Triton X-100 for 10 min and blocked with 5% bovine serum albumin in PBS for 1 h. Kv1.5 was labeled with mouse anti-Kv1.5 antibody (A3, sc-377110, Santa Cruz Biotechnology) and Alexa Fluor 488-conjugated goat anti-mouse secondary antibody (A11001, Invitrogen). Ub was stained with rabbit anti-Ub (U5379) and Alexa Fluor 594-conjugated donkey anti-rabbit secondary antibody (A21207, Invitrogen). The coverslips were mounted onto glass slides using Prolong Gold Antifade reagent. Images were acquired using the Quorum WaveFX-X1 spinning disc confocal system (Quorum Technologies Inc) at Queen’s University Department of Biomedical and Molecular Sciences Core Facility Centre. The signal intensities on the lines across the cells were plotted using ImageJ to show their cellular localizations.

### Reagents and antibodies

N-terminal specific mouse anti-Kv1.5 antibody (A3, sc-377110) was purchased from Santa Cruz Biotechnology Inc. The horse anti-mouse (#7076) and goat anti-rabbit (#7074) HRP-linked secondary antibodies were purchased from Cell Signaling Technology. TAK-243 was purchased from Cayman Chemical Company. Nystatin was obtained from Bioshop Canada. MEM, FBS, trypsin, sodium pyruvate, minimal essential amino acids, Lipofectamine 2000, Opti-MEM, G418, Alexa Fluor 488 and Alexa Fluor 594-conjugated secondary antibodies, ProLong Gold Antifade mountant, Texas Red X-conjugated wheat germ agglutinin were purchased from Thermo Fisher Scientific. Phorbol 12-myristate 13-acetate (PMA), rabbit anti-Ubiquitin (U5379), mouse anti-actin primary antibodies (A4700), and all chemicals/electrolytes used for electrophysiology were obtained from Sigma-Aldrich. The specificity of each antibody was validated by probing positive and negative protein samples as well as comparing them with other antibodies that probe different proteins. The BLUeye Prestained Protein Ladder (Bio-Helix) was purchased from FroggaBio. X-ray films were purchased from Fujifilm. Paraformaldehyde was obtained from Thermo Fisher Scientific.

### Statistical analysis

All data are expressed as mean ± standard deviation (SD). For box plots, original data points are superimposed. The line in the box represents the median, the small square represents the mean, and the bars represent SD. For summarized data of current-voltage relationships, current densities are calculated as peak current divided by the cell capacitances (pA/pF) and then plotted *versus* depolarizing voltages. Data were tested for normal distribution using the Kolmogorov-Smirnov test. For experiments with multiple groups, a one-way analysis of variance (ANOVA) with Tukey’s post-hoc test was used. For experiments between two groups, a two-tailed unpaired Student’s *t* test was used. A *p*-value of <0.05 is considered statistically significant.

## Data availability

All data are contained within the manuscript.

## Conflict of interest

The authors declare that they have no conflicts of interest with the contents of this article.
